# The Effect of Medium-Chain Triglycerides Oil on *Candida albicans* and *Streptococcus mutans* in Planktonic and Mucosal Models

**DOI:** 10.3390/antibiotics13121231

**Published:** 2024-12-20

**Authors:** Hiba Rashid Alyami, Yan Wu, Abdulwahab Aljughaiman, Ting Li, Abdullah Almulhim, Joseph M. Bliss, Jin Xiao

**Affiliations:** 1 Eastman Institute for Oral Health, University of Rochester Medical Center, Rochester, NY 14642, USA; hiba_alyami@urmc.rochester.edu (H.R.A.); yan_wu@urmc.rochester.edu (Y.W.); abdulwahab_aljughaiman@urmc.rochester.edu (A.A.); ting_li@urmc.rochester.edu (T.L.); abdullah_almulhim@urmc.rochester.edu (A.A.); 2 Dental Hospital, Imam Abdulrahman Bin Faisal University, P.O. Box 1982, Dammam 31441, Saudi Arabia; 3 Department of Stomatology, Union Hospital, Tongji Medical College, Huazhong University of Science and Technology, Wuhan 430042, China; 4 Department of Pediatrics, University of Rochester Medical Center, Rochester, NY 14642, USA; joseph_bliss@urmc.rochester.edu

**Keywords:** MCT oil, *C. albicans*, *S. mutans*, epithelial barrier, adherence, transmigration

## Abstract

Despite MCT oil’s potential antimicrobial benefits for gastrointestinal health, its effects on disrupting cariogenic pathogens on oral mucosal surfaces remain underexplored. This study evaluated the impact of MCT oil on the adhesion and invasion of *Candida albicans* and *Streptoccocus mutans* using planktonic and mucosal models. First, a planktonic model was used to assess the impact of various concentrations of MCT on the growth of *S. mutans* and *C. albicans*. Subsequently, a mucosal model was established by seeding TR-146 human buccal mucosal epithelial cells on a 3 µm porous transwell membrane, forming an epithelial barrier. MCT oil was then applied to the epithelial barriers in different durations (10, 30, and 60 min). Subsequently, *C. albicans* and *S. mutans* were introduced in the transwell and their adherence to the epithelial cells and their transmigration through the barriers was assessed using colony-forming unit counts and the barrier integrity was assessed by trans epithelial electrical resistance (TEER). Furthermore, cytotoxicity of MCT oil on mucosal cells was assessed by AlamarBlue assay. We found that higher MCT concentrations (90% and 100%) significantly inhibited *C. albicans* and *S. mutans* growth in planktonic conditions. Additionally, MCT oil reduced *S. mutans* adhesion to epithelial cells, highlighting its potential to interfere with bacterial attachment and colonization to oral mucosa. However, the oil had limited effects on *C. albicans* adhesion and transmigration. MCT demonstrated no cytotoxic effects on the viability of epithelial cells. The study findings highlight the potential benefits of MCT oil, particularly in oral bacterial inhibition, for oral health applications.

## 1. Introduction

Coconut oil, derived from mature coconut kernels, has gained widespread recognition for its health benefits [1,2,3]. Rich in medium-chain fatty acids, particularly lauric acid, coconut oil exhibits potent antimicrobial properties [4,5]. One prominent application is oil pulling, an ancient Ayurvedic practice involving swishing oil in the mouth to promote oral health [4,6,7]. Studies have demonstrated that coconut oil pulling effectively reduces *Streptococcus mutans* (*S. mutans*) counts in saliva, decreases plaque formation, and alleviates plaque-induced gingivitis [8,9,10,11]. Additionally, it shows comparable efficacy to chlorhexidine mouthwash in reducing bacterial counts, with the added benefit of causing less staining [9,12,13]. A randomized controlled trial further revealed significant plaque reduction within seven days of coconut oil pulling [6], highlighting its potential as an adjunct to oral hygiene.

Beyond its antibacterial properties, coconut oil demonstrates antifungal activity, particularly against *Candida albicans* (*C. albicans*), a common fungal pathogen in the oral cavity [8,14,15]. The antimicrobial effect is sustained, with significant reductions in both *S. mutans* and *C. albicans* observed within 24 h of contact [15]. A meta-analysis further confirmed the superior efficacy of coconut oil pulling over chlorhexidine in reducing *S. mutans* counts [16]. These findings support the use of coconut oil pulling as a safe, natural, and effective alternative for maintaining oral health.

MCT oil, a derivative of coconut oil, demonstrates potent antimicrobial properties attributed to its medium-chain fatty acids, including caprylic (C8) and capric (C10) acids [17,18,19]. Beyond gastrointestinal and biofilm studies, its impact on mucosal infections like candidiasis is underexplored. This study focuses on MCT oil’s potential to disrupt pathogenic interactions and protect mucosal integrity [18,20]. MCT oil has also demonstrated antifungal properties. A study found that dietary supplementation with MCTs reduced *Candida* colonization in the gastrointestinal tract of preterm infants, highlighting its potential to inhibit fungal growth [21]. This finding underscores MCT oil’s effects on the microbiota of mucosal surfaces and its potential for mitigating mucosal infections such as candidiasis.

Since oil pulling interacts directly with the mucosal surface, it is essential to address conditions like oral candidiasis, a fungal infection primarily caused by *C. albicans* [22,23]. Oral candidiasis, also known as thrush, occurs when *C. albicans* overgrows on mucosal surfaces, manifesting in acute, chronic, or Candida-associated forms [24]. Several factors contribute to its development, including impaired salivary function, diabetes, high-carbohydrate diets, smoking, denture use, and immunosuppressive conditions [22,25].

While *C. albicans* is typically a commensal organism, disruptions in the host microenvironment can trigger its transformation into a pathogenic state, leading to tissue invasion through virulence factors [26]. Diagnosis of oral candidiasis is primarily clinical, though laboratory analysis may sometimes be necessary [22,27]. Treatment involves topical or systemic antifungal medications, depending on severity [24]. Maintaining oral hygiene and ensuring early diagnosis are crucial for effective management, especially in immunocompromised individuals, where the infection may become systemic [28,29,30].

Emerging evidence highlights a synergistic relationship between *C. albicans* and *S. mutans* within oral biofilms, amplifying virulence and promoting caries development [31]. *C. albicans* and *S. mutans* interactions are not limited to dental biofilms but also play a role in mucosal infections, where synergistic interactions can amplify virulence and tissue invasion. Addressing these interactions on mucosal surfaces is critical for managing oral health conditions like candidiasis [32].

Dental caries, primarily caused by acidogenic bacteria such as *S. mutans* and *Lactobacillus*, result from dietary carbohydrate metabolism and subsequent acid production [33,34]. While biofilm formation plays a significant role in caries progression, its connection to bacterial-fungal interactions, particularly with *C. albicans*, underscores the need for broader preventive strategies [35,36]. Given the complex interactions between these pathogens and the link between oral and systemic health, early intervention is essential for preventing long-term complications [34].

Despite MCT oil’s promising antimicrobial properties, its role in disrupting the interaction between *C. albicans* and *S. mutans* on mucosal surfaces remains underexplored. While most studies focus on gastrointestinal health and dental biofilms, there is limited research on their impact on mucosal infections like oral candidiasis. This gap presents an opportunity to investigate MCT oil’s therapeutic potential in managing oral infections and preventing pathogenic interactions.

This study aims to evaluate the impact of MCT oil on the adhesion and invasion of *S. mutans* and *C. albicans* using planktonic and mucosal models. We hypothesize that MCT oil inhibits the pathogens’ adhesion and invasion, contributing to strategies for improving oral health and preventing infections like dental caries and oral candidiasis. The study assesses the cytotoxicity of MCT oil on mucosal cells to ensure it does not harm host tissues. Additionally, it examines the effect of MCT oil on adhesion at exposure times of 10, 30, and 60 min and evaluates the invasion potential of these pathogens after 18 and 24 h of incubation with and without MCT oil treatment.

## 2. Results

### 2.1. The Impact of MCT on Cariogenic Pathogens in the Single- and Dual-Species Planktonic Conditions

**Figure 1:**
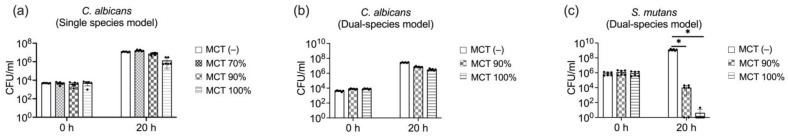
Effect of MCT on *C. albicans* and *S. mutans* in single- and dual-species planktonic models. (**a**) The growth of *C. albicans* cultured with varying concentrations of MCT. (**b**) The growth of *C. albicans* cultured with varying concentrations of MCT in the presence of *S. mutans*. (**c**) The growth of *S. mutans* cultured with varying concentrations of MCT in the presence of *C. albicans*. One-way ANOVA was used to compare the CFU counts (converted to natural log value). * A two-sided *p*-value which showed significant differences between MCT-treated (90% and 100% MCT) and untreated control groups was reported.

We first examined the impact of different MCT concentrations on the growth of *C. albicans* within a cariogenic planktonic model. After 20 h in a single-species condition, 90% and 100% MCT significantly inhibited *C. albicans* growth by 0.8 and 2.7 logs, respectively, while 70% MCT had no inhibitory effect (Figure 1a). Consequently, 90% and 100% MCT were selected for the dual-species model. Notably, despite an increase in *C. albicans* growth in the presence of *S. mutans* at 20 h, 90% and 100% MCT demonstrated inhibitory effects on both species. As shown in Figure 1b,c, *C. albicans* growth was reduced by 1.32 and 2.2 logs, while *S. mutans* growth was inhibited by an extraordinary 14.5 and 20.4 logs. These findings highlight the strong antimicrobial potential of high-concentration MCT in inhibiting both *C. albicans* and *S. mutans* in cariogenic environments.

### 2.2. The Impact of MCT on Viability of Oral Epithelial Cells

**Figure 2:**
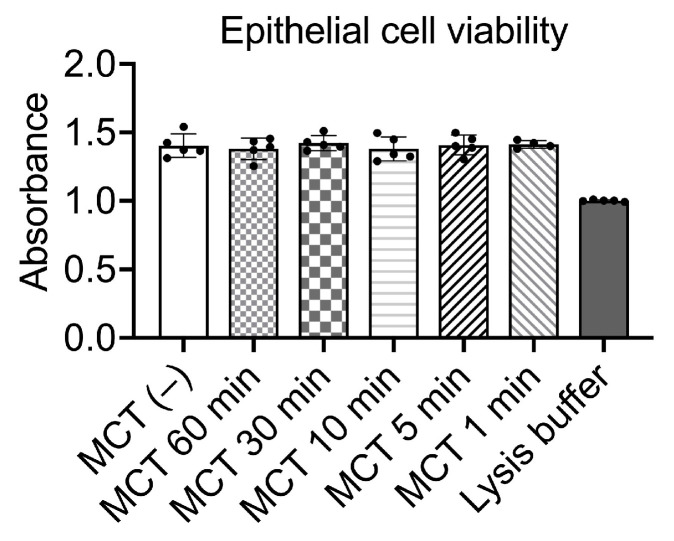
Viability of oral epithelial cells after varying exposure durations to MCT, as measured by alamarBlue assay. Oral epithelial cell viability was assessed after exposure to MCT for different durations. One-way ANOVA was used to compare the absorbance values. No significant difference between MCT-treated and untreated control groups was found when a two-sided *p*-value was reported.

We then evaluated the viability of oral epithelial cells following exposure to MCT to assess whether high-concentration MCT (100%) exhibits any cytotoxic effects. As shown in Figure 2, MCT applied to oral epithelial cells for varying durations, ranging from 1 to 60 min, showed no inhibitory effect on cell viability, demonstrating its high biocompatibility with human epithelial cells. These results suggest that 100% MCT does not compromise epithelial cell viability, further supporting its potential for safe therapeutic application.

### 2.3. The Impact of MCT on Adherence of C. albicans and S. mutans to Oral Epithelial Cells

**Figure 3:**
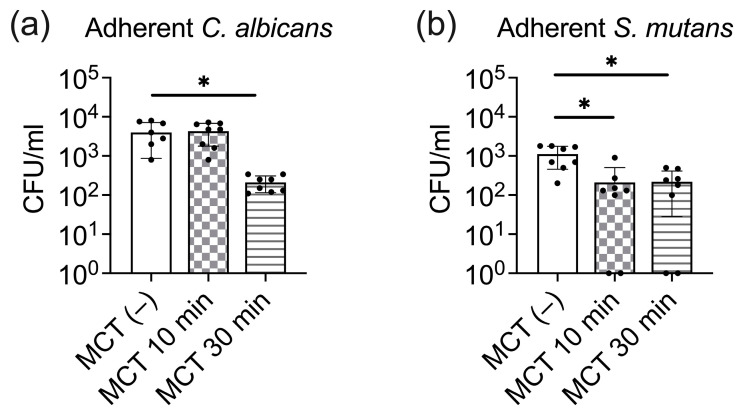
Effect of MCT on the adherence of *C. albicans* and *S. mutans* to oral epithelial cells. (**a**) Quantification of *C. albicans* adherence to epithelial cells, with and without MCT pre-treatment. (**b**) Quantification of *S. mutans* adherence to epithelial cells, with and without MCT pre-treatment. One-way ANOVA was used to compare the CFU counts (converted to natural log value). * A two-sided *p*-value which showed significant differences between MCT-treated and untreated control groups was reported.

To investigate the effect of MCT on the adherence of cariogenic pathogens to oral epithelial cells, we established a monolayer of epithelial cells on a porous membrane and applied 100% MCT to the apical surface. One-hour post-inoculation, *C. albicans* demonstrated an average adherence of 2.06 × 10^2^ CFU/mL to the epithelial layer, as shown in Figure 3a. MCT pre-treatment for 30 min significantly alter *C albicans* adherence levels (*p* < 0.05). Conversely, MCT 10 min treatment did not affect the adherence of *C. albicans*, as illustrated in Figure 3b, MCT pre-treatment markedly inhibited the adherence of *S. mutans*, resulting in statistically significant reductions of 2.8 and 2.6 logs (*p* < 0.05), respectively, indicating the potential of MCT as an anti-adhesive agent against this pathogen.

### 2.4. The Impact of MCT on Transmigration of C. albicans and S. mutans Through Oral Epithelial Barrier

**Figure 4:**
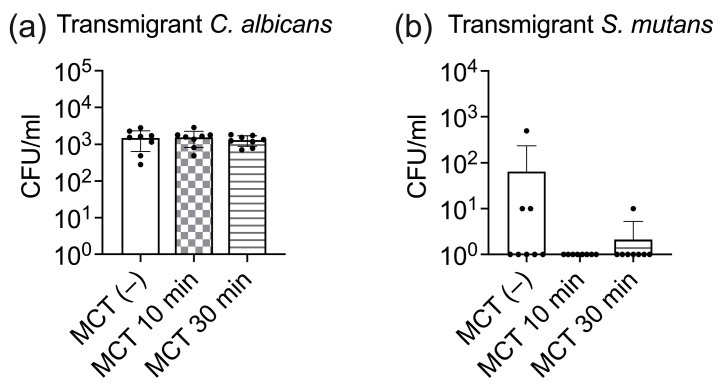
Effect of MCT on the transmigration of *C. albicans* and *S. mutans* through oral epithelial barrier. (**a**) Quantification of transmigrant *C. albicans* into the lower chamber, with and without MCT pre-treatment. (**b**) Quantification of transmigrant *S. mutans* into the lower chamber, with and without MCT pre-treatment. One-way ANOVA was used to compare the CFU counts (converted to natural log value). No significant difference between MCT-treated and untreated control groups was found when a two-sided *p*-value was reported.

To further elucidate the influence of MCT on cariogenic pathogens we assessed the effects of 100% MCT on *C. albicans* and *S. mutans* transmigration across the epithelial barrier. Eighteen hours post-inoculation, C. albicans transmigrated through the epithelial layer at an average rate of 1.46 × 10^3^ CFU/mL. MCT pretreatment for both 10 and 30 min did not significantly affect the transmigration of *C. albicans*. In contrast, *S. mutans* exhibited substantially lower transmigration rates than *C. albicans*, indicating a comparatively reduced invasive capacity. Furthermore, MCT treatment did not inhibit the transmigration of *S. mutans*, suggesting that MCT may not exert effects on the invasive behavior of this pathogen (Figure 4b).

### 2.5. The Impact of MCT on Oral Epithelial Barrier Integrity against C. albicans and S. mutans Infection

**Figure 5:**
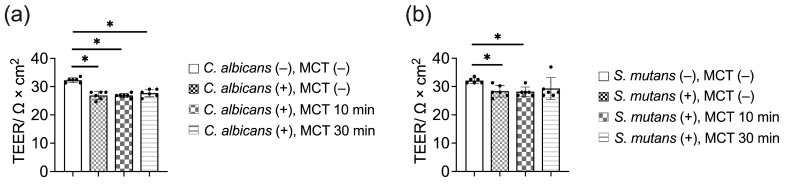
TEER (transepithelial electrical resistance) measurements of the oral epithelial barrier during *C. albicans* and *S. mutans* infection. (**a**) TEER measurements of the epithelial barrier during *C. albicans* infection, with and without MCT pre-treatment. (**b**) TEER measurements of the epithelial barrier during *S. mutans* infection, with and without MCT pre-treatment. One-way ANOVA was used to compare the TEER measurements. * A two-sided *p*-value which showed significant differences between MCT-treated and untreated control groups was reported.

Trans epithelial electrical resistance (TEER) measurements were utilized to assess the integrity and permeability of oral epithelial cell layers. TEER quantifies the electrical resistance across the cell monolayer, serving as an indicator of tight junction functionality and overall barrier integrity. Higher TEER values signify a more robust barrier, while reductions indicate compromised integrity. As shown in Figure 5, both *C. albicans* and *S. mutans* infections led to a significant decrease in barrier function compared to non-infected controls. Notably, MCT treatment for 30 min demonstrated a trend toward the restoration of barrier integrity, however, the barrier TEER value in *C. albicans* infection condition was still statistically significant different between the non-infection and MCT 30 min group (Figure 5a); while MCT 30min treatment restored the TEER value of the epithelial barrier to a non-significant different status when comparing to the non-*S. mutans* condition (Figure 5b). This result suggests a potential protective effect that warrants further investigation.

## 3. Discussion

This study evaluated the antimicrobial efficacy of medium-chain triglyceride (MCT) oil against *S. mutans* and *C. albicans* using planktonic and mucosal models. Results indicate that higher concentrations of MCT oil (90% and 100%) significantly inhibited the growth of *S. mutans* and *C. albicans* under planktonic conditions. Additionally, MCT oil reduced *S. mutans* adhesion to epithelial cells, highlighting its potential to interfere with microbial attachment and colonization. However, the oil had a limited effect on *C. albicans* adhesion and transmigration across epithelial layers, indicating that its antifungal efficacy may be limited in mucosal settings. These findings underscore the selective benefits of MCT oil, particularly in bacterial inhibition, for oral health applications.

Our findings align with previous literature supporting the antimicrobial efficacy of coconut oil and its derivatives against oral pathogens. Studies have shown that coconut oil pulling effectively reduces *S. mutans* counts, plaque formation, and plaque-induced gingivitis [8,9,11]. Similarly, Nandlal Bhojraj’s study reported that virgin coconut oil (VCO) mouthwash significantly reduced *S. mutans* counts in children, suggesting its suitability as a regular mouthwash [37]. The antibacterial effect of coconut oil concentrations has also been demonstrated in vitro, with higher concentrations (75%) yielding the largest inhibition zones and complete bacterial eradication [38].

These antibacterial effects extend beyond *S. mutans*, as both coconut oil and Nigella sativa oil inhibited *C. albicans* in studies by El-Sayed (2017) [15]. Furthermore, Lui Dwen Tjin [39] found that VCO at 25% concentration inhibited *C. albicans* growth within two days, showing comparable effectiveness to synthetic antifungals. Mukhtar’s research corroborated these findings, highlighting that activated VCO disrupts the fungal cell membrane, which suggests a promising antifungal mechanism [40]. However, our results suggest that although MCT oil inhibits fungal growth in planktonic models, it may be less effective in preventing *C. albicans* adhesion and transmigration across mucosal surfaces. This discrepancy may be attributed to structural differences between planktonic and mucosal environments, as well as complex host-pathogen interactions not captured in vitro [41].

The antibacterial findings in this study also align with research by Grecia Vásquez-Vereau [38], who demonstrated that increasing coconut oil concentrations led to larger inhibition halos against *S. mutans*. Additionally, the significant reductions in *S. mutans* adhesion observed in our study resonate with previous reports indicating that coconut oil and chlorhexidine mouthwash have similar efficacy in reducing bacterial counts, with coconut oil presenting the added advantage of causing less staining [9,12]. These results further highlight the potential of coconut oil and MCT oil as effective alternatives for managing bacterial biofilms, a key factor in oral infections.

The antifungal potential of MCT oil is further supported by findings from Arsenault et al. (2022) [21], who reported that dietary MCT supplementation significantly reduced *Candida* colonization in preterm infants, suggesting MCT’s broader antifungal utility. However, the limited impact of MCT oil on *C. albicans* adherence and transmigration in our study raises questions about its efficacy in preventing fungal biofilms on mucosal surfaces. These findings emphasize the need for additional research to explore the molecular mechanisms of MCT oil in microbial interactions, especially in mixed species systems where *S. mutans* and *C. albicans* synergistically enhance virulence [32].

Despite its promising antimicrobial effects, our study has several limitations that should be considered when interpreting the results. While in vitro models allowed for controlled experimentation, they do not fully replicate the complex microbial ecosystems present in the oral cavity, where interactions between *S. mutans*, *C. albicans*, and other microorganisms could influence outcomes differently in vivo. We acknowledge that the inclusion of a positive control, such as chlorhexidine, would provide valuable comparative data. Chlorhexidine is a widely used antimicrobial agent with established efficacy, making it a useful benchmark for evaluating new agents like MCT oil. However, the current study aimed to establish the standalone effects of MCT oil as an antimicrobial agent in both planktonic and mucosal models. Future studies will incorporate chlorhexidine and other oral care agents as positive controls to further evaluate the comparative efficacy and potential advantages of MCT oil, particularly its ability to minimize side effects such as staining or cytotoxicity. Additionally, the current study assessed cell viability using short-term exposure durations (1 to 60 min) to establish the immediate cytotoxic effects of MCT oil. While this provides critical insights into the safety of MCT oil for short-term applications, we agree that evaluating cell proliferation over longer intervals, such as 1, 3, 5, and 7 days, would provide valuable information on the potential for delayed cytotoxicity or impaired cell growth. Future studies will incorporate longer-term proliferation assays to comprehensively evaluate the impact of MCT oil on epithelial cell dynamics and ensure its suitability for extended use. This study represents an initial step in exploring the potential antimicrobial effects of MCT oil, focusing on single-species models to establish foundational data on its activity against *C. albicans* and *S. mutans*. We recognize the significance of using dual-species models to study the interactions between *S. mutans* and *C. albicans*, particularly given their synergistic behavior in biofilm formation and pathogenesis. However, the current study was designed as an exploratory investigation focusing on single-species models to establish baseline data on the antimicrobial effects of MCT oil. This approach allowed us to isolate and characterize the effects on each pathogen independently. Future research will build on these findings by incorporating dual-species models to evaluate the interaction between *S. mutans* and *C. albicans* under MCT oil treatment and better understand its potential to disrupt synergistic pathogenic behaviors. Additionally, the controlled laboratory environment lacks dynamic factors such as saliva flow, enzymatic activity, and immune responses, which may affect microbial behavior and impact the real-world relevance of our findings. Although our study assessed the effects of MCT oil on microbial adhesion and growth, it did not capture other aspects of pathogenicity, such as toxin production or resistance mechanisms, which could also play a role in oral infections. The study was limited by short-term observations, with experiments conducted over specific time points (10, 30, and 60 min for adhesion; 18 and 24 h for invasion), and longer-term research is needed to assess sustained effects. The current study focused on the short-term effects of MCT oil on bacterial adhesion and invasion to establish foundational data. While this provides critical insights into the immediate antimicrobial activity of MCT oil, we acknowledge that long-term evaluations are necessary to enhance clinical relevance. For example, preventive agents like mouthwash often require 7 to 14 days of daily application to achieve sustained effects. The time points selected for this study were based on the specific objectives of each experiment and relevant literature. For planktonic and adhesion assays, 20 h incubation was chosen to reflect a typical growth period for *S. mutans* and *C. albicans* under laboratory conditions, ensuring mature microbial cultures for analysis [42]. Transmigration assays used 18 and 24 h time points to capture early and intermediate stages of pathogen invasion through the epithelial barrier [43]. These time points align with previous studies investigating microbial dynamics in similar in vitro models and provide insights into both acute and sustained interactions between pathogens and host tissues. Future research will incorporate extended exposure times to assess the cumulative impact of MCT oil on bacterial and epithelial cell dynamics over clinically relevant timeframes. We agree that evaluating biofilm formation during transmigration assays would provide additional insights into microbial behavior. However, the current study focused on assessing the adherence and transmigration of *S. mutans* and *C. albicans* through the epithelial barrier. This approach was chosen to isolate the effects of MCT oil on adhesion and transmigration dynamics without the added complexity of biofilm structures. Future studies will incorporate biofilm evaluation to better understand the potential of MCT oil to disrupt biofilm formation during transmigration and its implications for managing oral mucosal infections. Furthermore, the lack of clinical trials limits the applicability of our results, highlighting the need for future studies involving human participants to validate the therapeutic potential of MCT oil for oral health.

In conclusion, this study highlights the potential of MCT oil as a natural agent for oral health applications, demonstrating significant antibacterial activity against *S. mutans*. However, its antifungal efficacy against *C. albicans* appears limited in mucosal settings, warranting further investigation. These findings support the inclusion of MCT oil and coconut-derived products as safe and effective alternatives to conventional oral care solutions. Future research should prioritize clinical trials to confirm these effects in vivo, while also exploring the impact of MCT oil on multi-species microbial communities and its role in preventing oral diseases. Expanding our understanding of the molecular mechanisms underlying these interactions will be essential to unlock MCT oil’s full therapeutic potential for oral health.

## 4. Materials and Methods

### 4.1. Bacterial Strains and Starter Preparation

The microorganisms used were *C. albicans* SC5314 and *S. mutans* UA159. Both strains were recovered from frozen stocks using YPD agar (BD Difco™, San Jose, CA, USA, 242720), and blood agar (TSA with sheep blood, Thermo Scientific™, Waltham, MA, USA), respectively. After 48 h of incubation, 3–5 colonies were inoculated into 10 mL broth for overnight incubation (37°C, 5% CO_2_). *C. albicans* was grown in YPD broth, and *S. mutans* in TSBYE (3% Tryptic Soy, 0.5% Yeast Extract Broth, BD Bacto™ 286220 and Gibco™ 212750) with 1% glucose. The next day, 0.5 mL of overnight cultures were transferred into fresh broth and incubated for 3–4 h to reach the mid-exponential phase, preparing the cultures for subsequent experiments.

### 4.2. Planktonic Model

The interactions between *Streptococcus mutans* and *Candida albicans* with MCT oil were assessed under planktonic conditions. Bacterial and fungal cultures were prepared by inoculating *S. mutans* UA159 and *C. albicans* SC5314 in brain–heart infusion (BHI) broth supplemented with 1% glucose and Yeast Peptone Dextrose (YPD) broth, respectively. Cultures were incubated at 37 °C under aerobic conditions with gentle shaking at 100 rpm until the mid-log phase. Optical density (OD600) was measured to standardize microbial suspensions to 10^5^ CFU/mL for *S. mutans* and 10^3^ CFU/mL for *C. albicans*.

MCT oil concentrations (0%, 10%, 30%, 50%, 70%, 90%, and 100%) were prepared in sterile tubes, each containing 1% glucose in a 10 mL total volume. The oil was vortexed briefly before addition to ensure even dispersion. Mono-species and dual-species models were prepared by adding microbial suspensions to the MCT oil solutions, followed by incubation in tryptic soy broth with yeast extract (TSBYE) and 1% glucose at 37 °C with 5% CO_2_ for 20 h. Tubes were placed on a shaker (100 rpm) during incubation to maintain homogeneous mixing.

Microbial growth was assessed by plating aliquots onto blood agar plates at predetermined time points (0, 2, 4, 6, and 20 h). Plates were incubated aerobically at 37 °C, and colony-forming units (CFUs) were enumerated to quantify microbial growth.

### 4.3. Epithelial Cell Culture and Transwell Model

The squamous carcinoma of buccal mucosa-derived epithelial cell line TR-146 (10032305) was purchased from Millipore Sigma (Darmstadt, Germany). TR-146 human buccal mucosal epithelial cells were thawed in a 37 °C water bath and transferred into tissue culture flasks (VWR^®^ 10062872, Radnor, PA, USA) with pre-warmed DMEM/F12 medium (Gibco™ 11320033) containing 10% *v/v* FBS (Gibco™ 16140071, Thermo Scientific™, Waltham, MA, USA). Cells were incubated at 37°C with 5% CO_2_, and the medium was replaced after 24 h. At 80–90% confluence, cells were subcultured using PBS and 0.25% trypsin (Millipore Sigma™ SM2001C), then reseeded at a 1:3 ratio. Expanded cells were seeded into 12-well Transwell inserts (VWR^®^10769-216, 12-well PET membrane, 3 μm pore size, 12 mm diameter) at a concentration of 1 × 10^5^ cells/insert to simulate the oral mucosal monolayer formation. After 14 days of incubation with media replacement every other day, the epithelial barrier was used for experimental procedures.

### 4.4. Cytotoxicity Assay

The cytotoxicity of 100% MCT oil (Nestlé Health Science, NJ, USA) on TR-146 buccal epithelial cells was evaluated using the AlamarBlue cell viability reagent (Invitrogen DAL1025) following the protocol from Thermo Fisher Scientific. TR-146 cells were seeded into 96-well plates (1 × 10^4^ cells/well) in DMEM with 10% FBS and incubated at 37 °C with 5% CO_2_ for 48 h to reach confluence. Cells were exposed to 100% MCT oil for 1, 5, 10, 30, and 60 min. Untreated wells served as negative controls, and lysis buffer-treated wells served as positive controls. After exposure, AlamarBlue reagent was added to each well, and the plates were incubated for 1–4 h at 37 °C. The reduction of resazurin to fluorescent resorufin by metabolically active cells was measured using a microplate reader, with the absorbance of the reagent monitored at 570 nm. This assay quantitatively measured cell viability and evaluated the cytotoxic effects of MCT oil to ensure its safety for subsequent adhesion and invasion assays.

### 4.5. Adhesion Assay

The adhesion of *S. mutans* or *C. albicans* to TR-146 cells was assessed following treatment with 100% MCT oil. Cells were grown in Transwell inserts for 14 days. MCT oil was applied to the apical side of the inserts for 10 and 30 min, followed by washing with PBS. Control wells did not receive MCT oil. After treatment, cells were inoculated with *S. mutans* (10^5^ CFU/mL) or *C. albicans* (10^3^ CFU/mL) and incubated for 1 h. Non-adherent bacteria were removed by washing, while adherent bacteria were scraped, resuspended, sonicated, and plated on blood agar for CFU counting after 48 h.

### 4.6. Transmigration Assay

The transmigration assay was conducted using HET-1A human esophageal epithelial cells grown on Transwell inserts (0.4 μm pore size; Corning, New York, NY, USA). Cells were seeded at a density of 5 × 10^5^ cells per insert in Dulbecco’s Modified Eagle Medium (DMEM) supplemented with 10% fetal bovine serum (FBS) and cultured at 37 °C with 5% CO_2_ until confluence. Before infection, the epithelial layer integrity was confirmed via transepithelial electrical resistance (TEER) measurements (see Section 4.7).

Microbial suspensions of *S. mutans* and *C. albicans* were prepared in TSBYE with 1% glucose and standardized to 1 × 10^6^ CFU/mL. For transmigration studies, 500 μL of microbial suspension was added to the apical chamber of each Transwell, and 1 mL of DMEM was placed in the basolateral chamber. Plates were incubated at 37 °C with 5% CO₂ for 18 or 24 h.

After incubation, 100 μL samples were collected from the basolateral chamber, serially diluted in phosphate-buffered saline (PBS) and plated on blood agar for *S. mutans* and Sabouraud agar for *C. albicans*. Plates were incubated aerobically at 37 °C for 24–48 h to enumerate CFUs, which were used to quantify transmigration through the epithelial barrier.

### 4.7. TEER Measurements

Transepithelial electrical resistance (TEER) was measured to evaluate the integrity of the epithelial monolayer before and after microbial application. An EVOM2 epithelial voltohmmeter (World Precision Instruments, Sarasota, FL, USA) equipped with STX2 electrodes was used for all measurements. Prior to each experiment, the instrument was calibrated according to the manufacturer’s guidelines using a blank Transwell insert containing only the culture medium.

For each measurement, 200 μL of pre-warmed phosphate-buffered saline (PBS) was added to the apical chamber, and 1 mL of PBS was added to the basolateral chamber. Electrodes were carefully inserted into the respective chambers, ensuring no contact with the epithelial layer. TEER values were recorded at baseline (pre-infection) and at 18- and 24 h post-infection. Resistance measurements were normalized to the surface area of the Transwell insert (0.33 cm^2^), and results were expressed in Ω·cm^2^. A drop in TEER of ≥20% from baseline was considered indicative of epithelial barrier disruption.

### 4.8. Statistical Analysis

To compare the abundance of *C. albicans* and *S. mutans* species in planktonic and mucosal models, the CFU/mL values were first converted into natural log values before analysis. Zero values were retained as zero. Normality tests were conducted to assess the data distribution among the variables including natural log-converted CFU/mL value, absorbance values, and TEER measurements. When data were normally distributed, the difference between groups was examined using one-way ANOVA for more than two groups followed by a post hoc test. Nevertheless, when data were not normally distributed, the Mann–Whitney U test was used to compare the results of the two groups, whereas the Kruskal–Wallis test was used to compare the results for more than two groups. Tests of statistical significance were two-sided with a significance level of *p* < 0.05. All analyses were performed in SPSS Version 24 (SPSS Statistics for Windows, Version 24.0; IBM, Armonk, NY, USA).

## Data Availability

All data generated or analyzed during this study are included in this article. Further inquiries can be directed to the corresponding author.
